# SNP-SNP Interaction Analysis on Soybean Oil Content under Multi-Environments

**DOI:** 10.1371/journal.pone.0163692

**Published:** 2016-09-26

**Authors:** Qingshan Chen, Xinrui Mao, Zhanguo Zhang, Rongsheng Zhu, Zhengong Yin, Yue Leng, Hongxiao Yu, Huiying Jia, Shanshan Jiang, Zhongqiu Ni, Hongwei Jiang, Xue Han, Chunyan Liu, Zhenbang Hu, Xiaoxia Wu, Guohua Hu, Dawei Xin, Zhaoming Qi

**Affiliations:** 1 College of Agriculture, Soybean biology Key Laboratory of the Ministry of Education, Northeast Agricultural University, Harbin, 150030, Heilongjiang, People’s Republic of China; 2 The Crop Research and Breeding Center of Land-Reclamation of Heilongjiang Province, Harbin, 150090, Heilongjiang, People’s Republic of China; 3 Crop Breeding Institute, Heilongjiang Academy of Agricultural Sciences, Harbin, 150086, Heilongjiang, People’s Republic of China; Harbin Medical University, CHINA

## Abstract

Soybean oil content is one of main quality traits. In this study, we used the multifactor dimensionality reduction (MDR) method and a soybean high-density genetic map including 5,308 markers to identify stable single nucleotide polymorphism (SNP)—SNP interactions controlling oil content in soybean across 23 environments. In total, 36,442,756 SNP-SNP interaction pairs were detected, 1865 of all interaction pairs associated with soybean oil content were identified under multiple environments by the Bonferroni correction with *p* <3.55×10^−11^. Two and 1863 SNP-SNP interaction pairs detected stable across 12 and 11 environments, respectively, which account around 50% of total environments. Epistasis values and contribution rates of stable interaction (the SNP interaction pairs were detected in more than 2 environments) pairs were detected by the two way ANOVA test, the available interaction pairs were ranged 0.01 to 0.89 and from 0.01 to 0.85, respectively. Some of one side of the interaction pairs were identified with previously research as a major QTL without epistasis effects. The results of this study provide insights into the genetic architecture of soybean oil content and can serve as a basis for marker-assisted selection breeding.

## Introduction

Soybean originates from China which is not only grain crop but also commercial crop. The content of protein accounts for 40% of soybean seeds, and the content of oil occupy 20%. Soybean is the largest oil seed crop in the world, which accounts for 57% of world oilseed production in 2015 (http://www.soystats.com) [1]. Oil content of soybean seeds is a complex quantitative trait governed by a number of genes mostly with small effects and was affected by the environment [2]. Till now, 188 QTL mapping research on soybean oil content were listed in the USDA Soybean Genome Database (http://www.soybase.org). Epistasis interaction is common and can cause cryptic genetic variation for quantitative traits [3]. Deriving genetic interaction networks from epistatic interactions between loci will enhance our understanding of biological systems that give rise to variation in quantitative traits [4]. Many recent studies have been performed regarding to epistatic interaction analysis, including investigations of agronomic and nitrogen acquisition traits in rice [5–9], and protein content, seed and pot traits, grain weight per plant in soybean [10–12], and proteins, oils, starches and fatty acids in maize [13–14], and grain protein, yield, and plant heights traits in wheat [15–17]. These studies, however, were based on the interactions of QTLs, which encompass more than a single marker. However, quantitative variation in phenotypes must result in multiple factor, therefore, interaction study designs have concentrated on estimating additive effects of single loci [18]. Because analyses of single loci can only partially investigate significant loci and may miss interactions between them, interaction analysis of single nucleotide polymorphisms (SNPs) is a better approach to elucidate genetic mechanisms [19], Goodman et al. [20] has explored the association between colon cancer and 94 SNPs, by the method, polymorphism interaction analysis in 2006. Dinu et al. [21] had discovered the Crohn’s disease genetics’ SNP-SNP interactions by logic regression. Lin et al. [22] studied five SNP-SNP interactions in three gene pairs associated with aggressive prostate cancer by SNP-SNP interaction network. Farzan et al. [23] found that RNASEL and MIR146A SNP—SNP interaction is a susceptibility factor for skin cancer. Li et al. [24] analyzed SNP—SNP interactions affecting abdominal fat weight by a linear mixed model.

Multifactor Dimensionality Reduction (MDR), inspired by the combinatorial partitioning method, can effectively reduce genotype predictors from *n* dimensions to one [25–29]. 2001, Ritchie et al. invented Multifactor dimensionality reduction which examines all possible SNP combination from a set of given SNPs and choose the combination that the best predicts risk by minimizing the classification error of cases and controls [26]. In addition, MDR analysis incorporates a cross-validation/permutation procedure to minimize the rate of false positive findings that may otherwise result from tests involving multiple variables or comparisons [25–27]. Although some recent studies on SNP interactions have exploited the MDR method [25–31], most of them has been focused on human and animal complex diseases and related traits. To date, no investigations of SNP interactions involving soybean-related traits have been conducted. This study is on the strength of a high-density genetic map for soybean which is based on specific length amplified fragment sequencing. We set the experiment in 23 environments in order to explore the stable locus across the multiple environments. MDR method was employed to analyze epistatic interactions between SNPs for oil content in soybean, the results on SNP interactions underlying genetic mechanisms of quantitative traits and may help improve soybean quality traits.

## Materials and Method

### Plant Materials

An F_2:10_–F_2:20_ population of 147 RILs was advanced by single-seed descent from crosses between two soybean cultivars: ‘Charleston’ (♀), (American cultivator) [32], and ‘Dongnong594’ (♂), (Northeast Agricultural University, Harbin, Heilongjiang, China). The 147 RILs were planted in three locations: Harbin (HRB; longitude 126°38′E, latitude 45°45′N) from 2002 to 2012; Hongxinglong (HXL; longitude 129°55′–134°35′E, latitude 45°35′–47°17′N) from 2007 to 2012; and Jiamusi (JMS; longitude 130°21′E, latitude 46°49′N) from 2007 to 2012.

The plants were arranged in a randomized complete block experimental design involving single-row plots (1-m length and 0.5-m width), with two and three replicates in 2002–2007 and 2008–2012, respectively. The timing and frequency of cultural management procedures used for the trials were in accordance with normal production practices for the respective environments.

### Measurement of Soybean Oil Content

Oil was analyzed from seeds obtained from five randomly harvested plants of each line per plot. The seed oil phenotypic data was analyzed using the FOSS Infratec TM1241 Grain Analyzer (FOSS Tecator AB, Hoeganaes, Sweden). The oil content of each RIL line was averaged from the data of two or three plot replicates. Two hundreds grams seeds of each replicate were used to analysis at a 13% moisture basis.

### Genotyping and Genetic Map Construction

SNP genotype data for the RIL population was detected based on the specific-locus amplified fragment sequencing (SLAF-seq) method following Qi et al. [33] and used to construct a high-density genetic map of 5,308 markers assigned to 20 linkage groups. The map spanned 2294.433 cM, with an average inter-marker distance of 0.43 cM. The percentage of gaps in which the distance between adjacent markers and markers is smaller than 5 cM (gap ≤ 5) is 99.84%.

### Interaction Analysis

To identify SNP × SNP effects in this study, we used the MDR method [25–31], The Pearson chi-squared module was used to assess significance (*p* < 0.001), with the selected optimization model based on the maximum Pearson chi-squared statistic combined with cross-validation [30].

The chi-square statistic measures the association between genotype (high-risk and low-risk group) and affection status (case and control group) in a two-way table. It is calculated as sum of the square of the difference between the observed and expected frequency in each combination, divided by the expected value, across all the combinations:

$${\text{x}}^{2}={\sum^{\;}}^{\;}\frac{{\left(observed-expected\right)}^{2}}{expected}$$

The calculation was based on the value of chi-square. Average Pearson chi-square of 10 time 10-fold cross-validation were used to select optimization model [30]. The p value was calculated referred the Bonferroni correction [34–35]. The epistatic interaction values and their contribution to genetic values and variance were calculated following the method proposed by Cheverud et al. [36]. Using contribution rate represent the phenotypic variation of the SNP—SNP interaction pair.

## Result

### Phenotypic Variation in the RIL Population under Multiple Environments

Oil content datas of the RIL population in 23 environments are shown in Table 1. The differences between the two parents were significant in parts of the 23 environments and the oil content of Charleston and Dongnong 594 ranged from 19.03 to 21.89% and from 18.96 to 21.83%, respectively. Oil content displayed a wide range of variation across the 23 environments, but the range of variation was narrow between different locations in the same year. The mean values of the RIL population ranged from 19.07 to 20.99%. The coefficient of variation ranged from 0.01 to 0.06, with a standard deviation of 0.36 to 1.39. Overall, the oil content of the RIL population was continuously distributed and consistent in all environments (Table 1).

**Table 1:** Oil content of the population and parents in 23 environments across 11 year.

Year and Environment	Charleston (%)	DongNong594 (%)	RIL lines					
			Mean (%)	Min (%)	Max (%)	SD	Kurtosis	Skewness
2002HRB	19.63	21.83	21.48	16.68	24.33	1.39	0.25	-0.36
2003HRB	19.27	19.68	20.46	17.90	22.61	0.91	-0.02	-0.08
2004HRB	19.03	19.26	19.28	17.87	20.92	0.60	-0.20	0.17
2005HRB	19.11	19.44	19.33	16.99	22.17	1.08	-0.30	0.37
2006HRB	20.30	21.20	20.86	19.05	21.90	0.50	0.46	-0.52
2007HRB	20.97	21.26	21.46	19.49	23.35	0.81	-0.10	0.07
2007HXL	21.09	21.63	22.40	18.99	25.88	1.36	-0.12	-0.01
2007JMS	20.65	20.98	20.99	18.64	22.76	0.77	0.44	-0.45
2008HRB	21.89	20.31	21.67	18.64	23.57	0.89	0.28	-0.43
2008HXL	21.80	19.89	21.02	18.64	23.59	0.82	0.83	0.58
2008JMS	21.77	20.73	22.19	19.38	24.13	0.90	0.67	-0.59
2009HRB	21.36	20.26	20.68	18.32	21.79	0.61	1.96	-1.15
2009HXL	21.24	18.96	20.42	17.85	22.28	0.67	1.37	-0.92
2009JMS	20.93	20.07	20.92	19.01	21.98	0.50	1.57	-0.59
2010HRB	21.21	21.42	21.37	19.73	22.03	0.41	2.63	1.27
2010HXL	21.73	21.42	21.56	20.14	22.36	0.39	1.27	0.69
2010JMS	21.51	21.49	21.49	19.09	23.20	0.48	4.64	0.80
2011HRB	20.56	21.14	21.33	20.01	22.51	0.40	0.55	-0.26
2011HXL	19.98	21.40	21.21	20.17	22.34	0.43	-0.22	-0.26
2011JMS	19.78	20.99	21.41	20.57	22.55	0.36	1.12	0.63
2012HRB	21.29	21.49	20.56	18.02	22.74	0.86	2.09	-0.42
2012HXL	20.73	21.16	19.07	15.51	20.86	0.43	1.85	-0.86
2012JMS	19.58	19.70	21.02	18.58	22.45	0.36	2.51	-0.66

### Pairwise Interaction Analysis

Analyze the oil content and genotype of 23 environments by the method of MDR. The selection criteria of SNP interaction pairs was the *p* <3.55×10^−11^ which was calculated based on the Bonferroni correction (Table 2). In total, the available interaction pairs have been detected in 12 of all environments, 36,442,756 interaction pairs were detected in all of the 12 environments. There were 5,612,873 pairs detected in 2006HRB, which were the most SNP interaction pairs. And the minimum number 24,689 pairs have been found in 2007HXL. 2005HRB, 2006HRB, 2007HRB, 2007JMS, 2008HRB, 2008HXL, 2009HXL, 2011JMS and 2012HXL were identified more than 1,000,000 SNP interaction pairs, 2004HRB, 2007HXL and 2008JMS were identified fewer than 100,000, and there were no available interaction pairs detected in the other 11 environments (Table 2).

**Table 2:** Interaction pairs numbers selected by MDR method in all environment (P<3.55×10^−11^).

Environment	The number of interaction pairs
2002HRB	NA^a^
2003HRB	NA^a^
2004HRB	87027
2005HRB	1098849
2006HRB	5612873
2007HRB	3391676
2007HXL	24689
2007JMS	4059216
2008HRB	2133998
2008HXL	2731592
2008JMS	60238
2009HRB	NA^a^
2009HXL	7432164
2009JMS	NA^a^
2010HRB	NA^a^
2010HXL	NA^a^
2010JMS	NA^a^
2011HRB	NA^a^
2011HXL	NA^a^
2011JMS	4113031
2012HRB	NA^a^
2012HXL	5697403
2012JMS	NA^a^
Total	36442756

^a^NA means no available interaction pairs in that environment.

**Fig 1:**
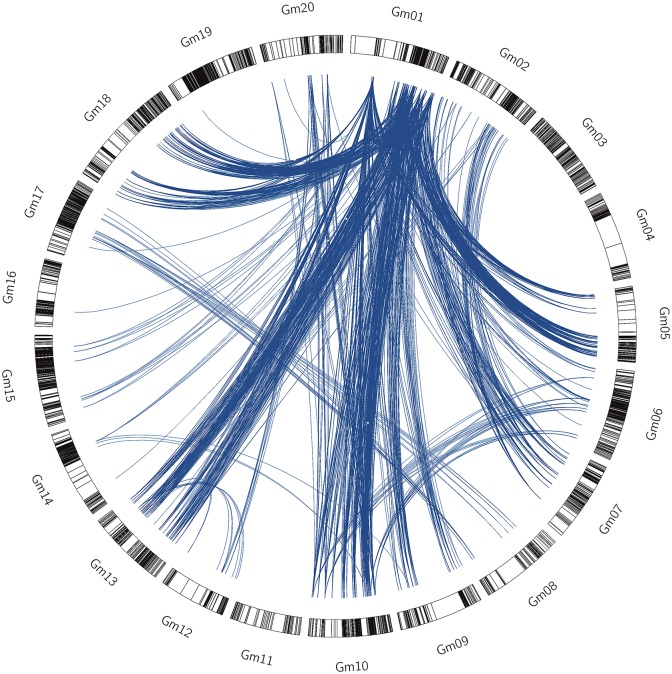
The circle represented the whole soybean genome. Blue lines represented epistatic interactions between two markers across eleven environment, the black lines represented epistatic interactions between two markers across twelve environment.

There were two and 1863 SNP interaction pairs were identified under 12 and 11 environments which have been detected the available interaction pairs (Fig 1). Among the 20 linkage groups, most of stable interaction pairs were from Gm01 to others, including Gm01, Gm05, Gm06, Gm07, Gm08, Gm09, Gm10, Gm13, Gm18 and Gm20. Two SNP interaction pairs detected across 12 environments showed the interaction effect between Gm01 (Mark673223) and Gm05 (Mark310931, Mark312588), furthermore, 1863 SNP interaction pairs detected across 11 environments showed the strong interaction region at Gm01, Gm05, Gm06, Gm07, Gm09, Gm10, Gm13 and Gm18 (Fig 1). These 1865 SNP interaction pairs were detected stable across environments which almost 50% of all, so we saw them as the stable interaction pairs and used for next step calculation.

### Epistatic Value and Contribution Rate Analysis

**Fig 2:**
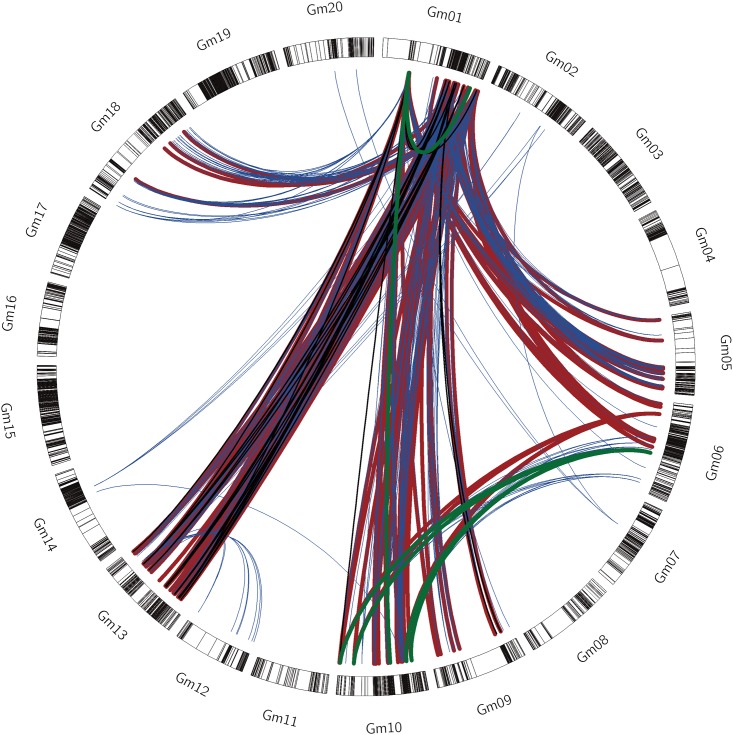
The circle represented the whole soybean genome. Blue lines indicate epistatic interactions across 2 environments, red lines indicate epistatic interactions across 3 environments, black lines indicate epistatic interactions across 4 environments, green lines indicate epistatic interactions across 5 environments.

The epistatic interaction values and their contribution to genetic values and variance were screened by the two way ANOVA test for each environment at significant level of *p*<0.01. In total, there were 560 of 1865 interaction pairs were screened, 4, 23, 99 and 344 SNP interaction pairs were detected as stable and effective interaction pairs across 5, 4, 3 and 2 environments respectively (Fig 2), which similar as the environment-universal QTLs, defined as QTLs associated with traits of interest in at least one macro-environment (i.e., year-location combination) [37, 38], here, it could be the environment-universal SNP interaction pairs, and there were no significance for the other SNP interaction pairs. Epistatic value and contribution rate data of stable interaction pairs across all environments are shown in S1 Table. Epistasis value was ranged from 0.01 to 0.89 at the p<0.01 level. Contribution rates of epistatic pairs were ranged from 0.01 to 0.85 at the p<0.01 level (S1 Table).

A compairson of the environmentally stable SNP interaction pairs uncovered in this study with those detected in previous research revealed none that matched completely. In a few cases, however, a member of an interaction pair matched a main effect QTL not previously known to participate in an interaction. Some regions on Gm01 15.0Mb-15.3Mb, 28.8Mb-38.0Mb have been mapped as major QTL fragments by Hyten et al. [39], from 40.7Mb to 41.0Mb has been detected by Specht et al. [40], 42.0Mb-43.3Mb has also been found by Eskandari et al. [41]. On Gm02 (34.6–34.8Mb) has been mapped as major QTL fragments by Qi et al. [42]. Some regions on Gm05, 1.3–1.6Mb, 27.6–27.8Mb, 31.2–32.0Mb, 33.2–34.8Mb and 37.6–39.5Mb all have been detected by Wang et al. [43], Mansur et al. [44], Lark et al. [45], Brummer et al. [46] and Liang et al. [47] respectively. Some regions on Gm06 from 1.2Mb to 1.5Mb, 14.4Mb, 21.2–29.0Mb, 24.7Mb, 37.8Mb and 46.7Mb have been mapped as major QTL fragments by Wang et al. [43], Hyten et al. [39], Chen et al. [48], Reinprecht et al. [49], Kim et al. [50], Qi et al. [42] respectively. On Gm09, 1.0Mb, 3.9Mb and 5.7Mb have been detected by Mansur et al. [44]. Wang et al. [43] have found from 8.2 to 9.1Mb and 41.1–42.8Mb as major QTL fragments. From 31.0Mb to 33.4Mb has been detected by Eskandari et al. [41]. Some regions on Gm10 from 10.2Mb to 13.7Mb and 46.2Mb have been detected by Panthee et al. [51] and Li et al. [52] respectively. The region on Gm12 10.8–14.0Mb and 35.0Mb have been found by Shibata et al. [53] and Eskandari et al. [41] respectively. On Gm13, 6.1–6.7Mb has been mapped as major QTL fragments by Qi et al. [42]; 27.1–28.3Mb has been detected by Specht et al. [40], 29.0–30.7Mb and 32.2–33.6Mb have been found by Eskandari et al. [41] and 31.3–31.5Mb has been found by Rossi et al. [54]. On Gm14 Eskandari et al. [41] and Liang et al. [47] have detected 32.2Mb and 35.9Mb. Some regions on Gm18, 16.7–18.3Mb, 41.66Mb, 46.2–57.6Mb, 50.0–52.7Mb and have been mapped as major QTL fragments by Reinprecht et al. [49], Wang et al. [43], Lee et al. [55] and Brummer et al. [46]. On Gm20, 24.6Mb and 36.5Mb have been detected by Shibata et al. [53] and Reinprecht et al. [49] respectively.

## Discussion

Soybean oil content is an important quantitative trait under complex genetic control. Understanding the oil content locus interaction mechanisms could help us effectively increase the oil content in soybean. In this study, MDR method was employed to identify the stable loci controlling oil content in soybean that is based on a high-density genetic map, which has been constructed by specific-locus amplified fragment sequencing (SLAF-seq) with 5308 markers on 20 linkage groups and the map was 2294.43 cM in length, with an average distance of 0.43 cM between adjacent markers [33].

Epistasis is a biochemically plausible feature of the genetic architecture of quantitative traits. So, epistasis causes hidden quantitative genetic variation in natural populations and could be responsible for the small additive effects [4]. In quantitative genetics, epistasis refers to any statistical interaction between genotypes at two or more loci [56–57]. The role of epistasis in the genetic architecture of quantitative traits has been controversial since early formulations of quantitative genetic theory [58–59]. Numerous methods to detect epistatic interactions between SNPs have been published, such as exhaustive algorithms [60] and MDR [25–33], regression [61–63], heuristic [64–65] and mutual information [66], among others [60, 67–70].

Currently, many studies are based on multifactor dimensionality reduction (MDR) focus on the complex human diseases and animal-related traits. Guia et al. [71] reveals interaction of important gene variants involved in allergy by MDR. Su et al. [72] applied the multifactor dimension reduction method to analyze gene-gene and gene-environmental interactions of childhood asthma. Gui et al. [73] detected gene-gene interactions with application to the genetic analysis of bladder cancer susceptibility with multifactor dimensionality reduction method. Cho et al. [74] using MDR method find out that a two-locus interaction genes among 23 loci in the candidate genes of Type 2 diabetes. Julia et al. [75] suggest that 13 genes is associated with synovial fibroblast response to rheumatoid arthritis proinflammatory stimulus by MDR. Many studies using the MDR method have focused on complex human and animal disease-related traits to identify the epistatic interactions [25–31, 71–75], to our knowledge, no study have applied MDR to analyze soybean quantitative traits. In this study, we identified 1865 stable SNP interactions pairs associations with soybean oil content under multi-environments, successfully applied MDR to the plant research. Among 1865 SNP interactions pairs, 2 pairs were detected stable across 12 environments, 1863 pairs were detected stable across 11 environments. MDR method can improve the analysis efficiency as an analysis method based on haplotype, which is a good choice for genome-wide interaction analysis. Nevertheless, this method also has some restrictions. First, it does not have a way to adjust for covariate effects such as age, gender and smoking status, an often necessary step to obtain an unconfounded SNP interaction outcome association [76]. Second, if there are too many covariates, this approach may over fit the data and sometimes fail due to limited sample size [77].

The present study has opened the door for further research on soybean quantitative trait integration analysis. On the one hand, the SNP-SNP interaction of hot region which were identical with some published major QTLs, however, those major QTLs were not shown the interaction each other previously. On the other hand, the candidate major region could be found as only one site of the pair was aligned with the published QTL. Based on this research, the markers of those hot region could be developed, and used for breeding selection process, furthermore, the candidate genes of hot region could be identified and to analysis the interaction of genes in next step. Future efforts based on the uncovered epistatic interaction values and their contribution to genetic values can be applied for the identification of key loci with higher phenotypic contributions, the prediction of phenotype and genotype design modules with epistatic values, and to provide direction for high-quality soybean breeding.

## Supporting Information

S1 Table Epistatic value and contribution rate data of stable interaction pairs across 11 environments. NS means no signification value in that environments, epistatic value indicates the epistatic effects value of the SNP—SNP pairs, contribution rates indicates the phenotypic variation of the SNP—SNP interaction pairs.
(XLSX)
