# Correction: SNP-SNP Interaction Analysis on Soybean Oil Content under Multi-Environments

**DOI:** 10.1371/journal.pone.0169222

**Published:** 2016-12-21

**Authors:** The PLOS ONE Staff

The following information is missing from the Funding section:

The National Natural Science Foundation of China: grants 31471516 and 31271747 to Qingshan Chen; grant 31401465 to Hongwei Jiang; grant 31400074 to Dawei Xin; and grant 31501332 to Chunyan Liu. New Century Excellent Talent Training Plan of Heilongjiang Province Ordinary Institutions of Higher Learning, grant 1252-NCET-004, to Qingshan Chen. Natural Science Foundation Key Program of Heilongjiang Province of China, grant ZD201213, to Qingshan Chen. Qingniancaijun project of Northeast Agricultural University, grant 518062, to Zhaoming Qi. New Century Excellent Talents in University of Ministry of Education, grant NECT-1207-01, to Qingshan Chen. Key Technologies Research and Development Program of China during the Twelfth Five-year Plan Period, grant 2011BAD35B06-1, to Qingshan Chen. Modern Agricultural Industry Technology System, grant CARS-04-02A, to Qingshan Chen. China Postdoctoral Science Foundation, to Zhaoming Qi. Colleges and universities in Heilongjiang Province of the Cheung Kong Scholars backup support program, grant 2014CJHB004, to Qingshan Chen. Harbin good foundation for leaders of disciplines, grant 2014RFXXJ012, to Qingshan Chen. SIPT Project of Northeast Agricultural University: grant 201610224146 to Zhongqiu Ni; grant 201410224111 to Leng Yue; and grant 201510224016 to Shanshan Jiang.

The publisher apologizes for the errors.
